# Biological characteristics and prognosis of acute myeloid leukemia in elderly patients

**DOI:** 10.3389/fgene.2025.1524177

**Published:** 2025-04-04

**Authors:** Zhe Chen, Lina Ding, Jieni Yu, Jing Jin, Zhijian Zhang, Jiaping Fu, Pan Hong, Leihua Fu

**Affiliations:** Department of Hematology, Shaoxing People’s Hospital, Shaoxing, China

**Keywords:** elderly AML, gene mutation, chromosomal aberrations, prognosis, retrospective

## Abstract

**Background:**

Our study aimed to investigate the effects of chromosomal aberrations and genetic mutations of elderly individuals diagnosed with AML and determine its prognostic significance.

**Methods:**

We retrospectively collected data over nearly 7 years from our hospital, encompassing 90 cases of elderly AML patients. Baseline information of patients was gathered and followed up, and statistical analysis was conducted using SPSS 25.0.

**Results:**

Among the 90 elderly non-M3 AML patients, 56 (62.2%) exhibited multiple gene mutations, with 9 (10%) patients displaying five or more gene mutations. The incidence of *NPM1* mutation was significantly higher in patients with normal karyotypes compared to those with abnormal karyotypes (P = 0.001). Patients with *FLT3*, *ASXL1*, or *TP53* mutations displayed lower rates of CR compared to wild-type counterparts. Kaplan-Meier analysis revealed that *TET2* mutation (P = 0.0474), *FLT3-ITD* mutation (P = 0.0364), *TP53* mutation (P = 0.0031), and 17p abnormality (P = 0.00285) were predictive of shorter OS. *TP53* mutations (P = 0.0440), 17p abnormalities (P = 0.0272), 7q abnormalities (P = 0.0174), and complex karyotypes (P = 0.0447) were associated with shorter RFS.

**Conclusion:**

Our findings suggest that elderly AML patients exhibit distinctive genetic profiles, and favorable prognosis genes do not seem to apply to elderly AML patients.

## Introduction

Acute myeloid leukemia (AML) represents a clonal malignant disorder arising from hematopoietic stem and progenitor cells. It manifests primarily through symptoms such as anemia, hemorrhage, infection, and extramedullary organ infiltration, progressing rapidly. While AML can affect individuals across all age groups, it predominantly occurs among the elderly, with a median age of 68 years at diagnosis, and around two-thirds of patients being over 55 years old (Sasaki et al., 2021). Recent publications, such as The Lancet, have highlighted a global mortality of over 80,000 AML-related deaths annually, a number anticipated to double within the next 20 years (Foreman et al., 2018). The 5-year relative survival rate for AML patients internationally stands at a mere 30.5%. Notably, the estimated 5-year survival rates indicate a significant difference among age groups: 62% for patients younger than 50 years old, 37% for those aged 50 to 64, and a starkly lower 9.4% for individuals diagnosed at 65 years and older (Lewis-thames et al., 2022).

Elderly AML patients are more difficult to treat than young patients, given the rising prevalence of elderly populations, addressing the treatment strategies for elderly AML patients holds paramount importance. Elderly AML patients tend to exhibit more unfavorable cytogenetic or molecular genetic abnormalities compared to younger counterparts (Papaemmanuil et al., 2016). While the incidence of AML might be linked to specific gene mutations, there remains a lack of evidence-based guidelines or consensus standards specifically tailored for elderly AML patients. As a result, this article primarily aims to investigate the association between cytogenetic or molecular genetic factors and the clinical characteristics and prognosis of elderly AML patients through retrospective analysis. By doing so, it seeks to evaluate patient prognoses more accurately and provide improved guidance for clinical management.

## Methods

### Patients

We conducted a retrospective analysis involving 90 newly diagnosed elderly AML (non-M3) patients at Shaoxing People’s Hospital between June 2016 and April 2023 (for specific details, please refer to the data file). The diagnostic criteria for elderly AML adhered to the updated WHO classification criteria in 2022 (Khoury et al., 2022). Follow-up data were collected until September 2023. This study received approval from our hospital’s ethics committee. Table 1 outlines the clinical characteristics of the patients.

**TABLE 1:** Clinical characteristics.

Characteristics	Statistics
Median age (years old)	67 (60–87)
Gender ratio (male/female)	47/33
Median BM blasts (%)	60 (20.5–91)
Median WBC count (×109/L)	4.01 (0.24–410.04)
Median hemoglobin (g/L)	77 (43–159)
Median platelet (×109/L)	48.5 (0–991)
Median LDH (U/L)	279.05 (99.4–2235.4)
FAB-classification	
M0	1 (1.1%)
M1	4 (4.4%)
M2	30 (33.3%)
M4	16 (17.8%)
M5	26 (28.9%)
M6	1 (1.1%)
Unclassified	12 (13.4%)
Response to chemotherapy	
CR/NR	54/36
Induction chemotherapy	
Standard-intensive chemotherapy	30 (33.3%)
Low-intensive chemotherapy	49 (54.5%)
Chemotherapy plus transplantation	11 (12.2%)
ELN risk assessment	
Favorable-risk	26 (28.9%)
Intermediate-risk	28 (31.1%)
High-risk	36 (40%)

### Cell genetics and gene mutation detection

For patients newly diagnosed with AML, bone marrow specimens were obtained from the posterior iliac spine before or during the initial diagnosis, yielding 5 mL of bone marrow fluid. These specimens were cultured in 20% calf serum for 24 h, followed by R-banding karyotype analysis. Monosomal karyotype (MK) was defined as the presence of at least two autosomal monosomies or one autosomal monosomy combined with at least one structural abnormality, excluding specific AML-RGA translocations [t (15; 17), t (8; 21), inv/t (16)]. Highly abnormal complex karyotype (CK) was defined as the presence of five or more unrelated chromosomal abnormalities.

Additionally, we utilized second-generation myeloid tumor gene sequencing methods (whole genome sequencing) to detect mutations in specific genes, including *TET2*, *DNMT3A*, *NPM1*, *FLT3*, *ASXL1*, *IDH2*, *NRAS*, *NF1*, *IDH1*, *TP53*, *RUNX1*, *KIT*, *BCORL1*, *PHF6*, *BCOR*, *KRAS*, *CEBPA*, *EZH2*, *CBL*, *U2AF1*, *ETV6*, *PIGA*, *WT1*, *PTPN11*, *SRSF2*, *STAG2*, *ZRSR2*, *JAK2*, *SF3B1*, and *SETBP1*.

### Induction chemotherapy

The preferred standard for intensive chemotherapy involves a 7 + 3 regimen with daunorubicin or idarubicin, using Ara-C at 100–200 mg/m^2^ per day for 7 days, combined with IDA at 12 mg/m^2^ per day for 3 days or DNR at 60–90 mg/m^2^ per day for 3 days.

The regimen includes low-intensity Venecola chemotherapy (100 mg on Day 1, 200 mg on Day 2, and 400 mg from Day 3 to Day 28) combined with azacitidine (75 mg/m^2^/day for 7 days) or decitabine (20 mg/m^2^/day for 5 days) administered every 28 days.

### Efficacy evaluation

Overall survival (OS) was defined as the duration from diagnosis until the conclusion of follow-up, transplantation, or death, while relapse-free survival (RFS) referred to the period from the initial complete remission to relapse, death, or the last follow-up. The assessment of efficacy aligned with NCCN guidelines (Pollyea et al., 2021). Remission (CR) was defined by bone marrow containing ≤5% blasts and normal maturation across all cell lines. Relapse was identified if blasts exceeded 5% subsequent to achieving CR.

### Statistical analysis

Analysis of the data’s normality or homogeneity of variance within each group was performed using SPSS 25.0 software. The Chi-square test was applied to compare categorical data between two groups, while one-way analysis of variance (ANOVA) was utilized for comparing data across multiple groups. Kaplan-Meier survival analysis and the Log-rank test were employed for inter-group comparisons. Indicators exhibiting significance in single-factor analysis underwent COX regression analysis for multifactorial examination. Results were deemed statistically significant when P < 0.05.

## Results

### The characteristics and clinical features in elderly patients with AML cytogenetics analysis

In our retrospective analysis of 90 elderly AML patients (excluding M3 subtype), comprising 52 males and 38 females, with a median age of 67 years (range: 60–87), with a median BM blasts of 60% (range:20.5–91), with a median WBC count of 4.01 × 109/L (range:0.24–410.04), with a median hemoglobin count of 77 g/L (range:43–159), with a median platelet count of 48.5 × 109/L (range:0–991). Table 1 outlines the clinical characteristics of the patients.

All patients underwent karyotyping. Chromosomal abnormalities were detected in 40 individuals (44.4%, 40/90). Among these abnormalities, CK was the most prevalent, observed in 20 cases (22.2%, 20/90), characterized by a highly abnormal chromosomal pattern (5 or more chromosomal abnormalities). MK and 7q-abnormalities were identified in 8 cases each (8.9%, 8/90), while +8, 5q-, and 17p abnormalities were present in 7 cases each (7.8%, 7/90).

**FIGURE 1:**
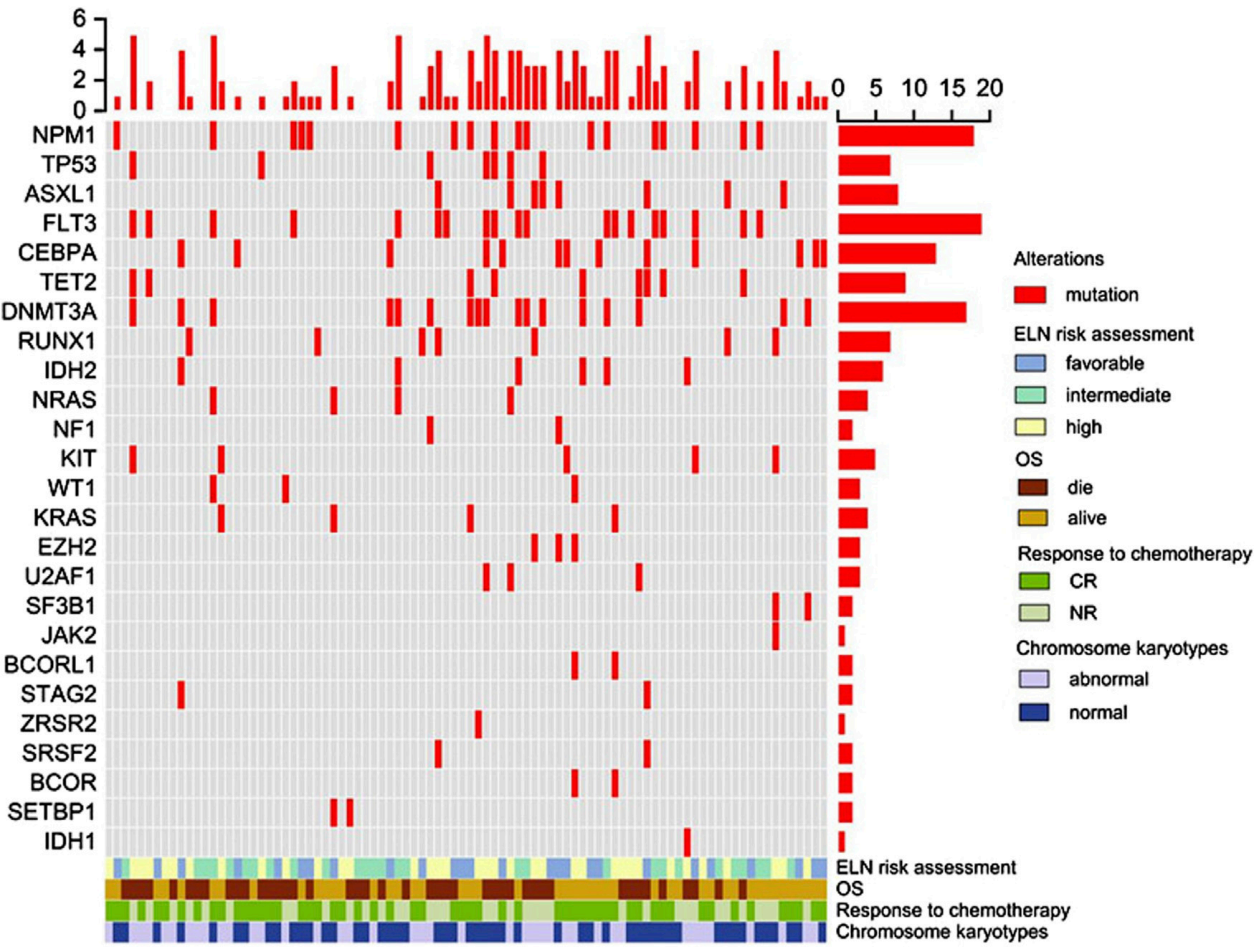
Overview of gene mutations, clinical features, and overall survival in elderly AML. Each column represents an individual patient, and the presence of the mutation is indicated in red. The upper panel shows the gene mutation, and the lower panel shows the clinical features and overall survival. Abbreviations: CR, complete remission; NR, no response; OS, overall survival.

Furthermore, all patients underwent gene mutation detection, revealing multiple gene mutations in 56 individuals (62.2%, 56/90), with 9 patients (10%, 9/90) exhibiting 5 or more gene mutations. *FLT3-ITD* mutation was the most frequent, identified in 19 patients (21.1%, 19/90), followed by *NPM1* mutation in 18 patients (20%, 18/90), and *DNMT3A* mutation in 17 patients (18.9%, 17/90). Other observed mutations included *CEBPA* in 14 cases (14.4%, 14/90), *TET2* in 13 cases (14.4%, 13/90), *ASXL1* and *RUNX1* in 8 cases each (8.9%, 8/90), *TP53* and *IDH2* in 7 cases each (8.9%, 7/90), *KIT* in 6 cases (6.7%, 6/90), and *NRAS* in 4 cases (4.4%, 4/90). Detailed information regarding specific gene mutations is depicted in Figure 1.

### Association of gene mutations with chromosome karyotype and CR

We conducted an evaluation focusing on single gene mutations concerning remission rates in elderly AML patients. Out of the 90 elderly patients assessed, 54 individuals (60%) attained CR following one or more courses of induction chemotherapy. These patients were categorized into CR and NR (non-remission) groups. Our findings indicated significantly lower CR remission rates among patients with *FLT3* (36.8% vs. 66.2%, P = 0.02), *ASXL1* (25% vs. 63.4%, P = 0.034), or *TP53* mutations (14.3% vs. 63.9%, P = 0.010) compared to those with wild-type genes.

We further investigated the relationship between gene mutations and chromosomal abnormalities in elderly AML patients. Among the 90 elderly AML patients evaluated, 40 individuals exhibited abnormal chromosome karyotypes, segregated into normal karyotype and abnormal karyotype groups. Our analysis revealed a significant variation in the distribution of *NPM1* mutations concerning different chromosome karyotypes (P = 0.001). Notably, the frequency of *NPM1* mutation was higher in patients with a normal karyotype (32%, 16/50) compared to those with an abnormal karyotype (5%, 2/40). For specific details, please refer to Table 2.

**TABLE 2:** Association of genetic mutations with clinical features of elderly AML patients.

Mutation		Chromosome karyotype normal/abnormal	Response to chemotherapy CR/NR
*FLT3-ITD*	Mutant vs. wild	14/5 vs. 36/35	7/12 vs. 47/24
	p value	0.073	0.020
*NPM1*	Mutant vs. wild	16/2 vs. 34/38	11/7 vs. 43/29
	p value	0.001	0.914
*DNMT3A*	Mutant vs. wild	11/6 vs. 39/34	10/7 vs. 44/29
	p value	0.399	0.912
*CEBPA*	Mutant vs. wild	9/4 vs. 41/36	10/3 vs. 44/33
	p value	0.283	0.178
*TET2*	Mutant vs. wild	7/2 vs. 43/38	4/5 vs. 50/31
	p value	0.157	0.315
*ASXL1*	Mutant vs. wild	4/4 vs. 46/36	2/6 vs. 52/30
	p value	0.740	0.034
*RUNX1*	Mutant vs. wild	3/4 vs. 47/36	2/5 vs. 52/31
	p value	0.481	0.077
*TP53*	Mutant vs. wild	2/5 vs. 48/35	1/6 vs. 53/30
	p value	0.135	0.010
*IDH2*	Mutant vs. wild	5/1 vs. 45/39	4/2 vs. 50/34
	p value	0.156	0.730
*KIT*	Mutant vs. wild	1/4 vs. 49/36	2/3 vs. 52/33
	p value	0.100	0.348

### The clinical features in elderly patients with AML prognosis impact analysis

We conducted an evaluation focusing on clinical features in elderly AML patients. In the cohort of 90 patients undergoing chemotherapy, 30 patients were treated with standard-intensive chemotherapy (33.3%), 49 patients were treated with low-intensive chemotherapy (54.5%). 54 patients achieved complete remission (60%), while 36 patients (40%) did not respond favorably to treatment. Subsequently, 11 patients (12.2%) received allogeneic transplantation therapy (see Table 1).

Kaplan-Meier analysis unveiled that clinical features were associated with OS. Notably, including not achieving CR (P = 0.00), low-intensive chemotherapy (P = 0.043), and high-risk group (P = 0.039), were predictive of shorter OS (see Table 3).

**TABLE 3:** Univariate analysis of the clinical features in elderly patients with AML.

Factors	n	p value
Response to chemotherapy (NR vs. CR)	36/54	0.00
Gender ratio (male/female)	52/38	0.316
Induction chemotherapy	30/49/11	0.043
ELN risk assessment	26/28/36	0.039
initial platelet count of ≤20 × 10^9^/L	79/11	0.688
initial Hb count of ≤60 g/L	79/11	0.640

### The biological characteristics in elderly patients with AML prognosis impact analysis

At the conclusion of the follow-up period, 47 patients (52.2%) were still alive. The median OS was 15.7 months (ranging from 1.5 to 86.9 months), and the median RFS was 13.8 months (ranging from 1.5 to 81.4 months). In the group of 19 AML patients with the *FLT3-ITD* mutation, 7 are alive (36.8%, 7/19). Among the 18 AML patients with the *NPM1* mutation, 10 are still living (55.5%, 10/18). In the 17 AML patients with the *DNMT3A* mutation, 11 remain alive (64.7%, 11/17). For specific details, please refer to Figure 1.

**FIGURE 2:**
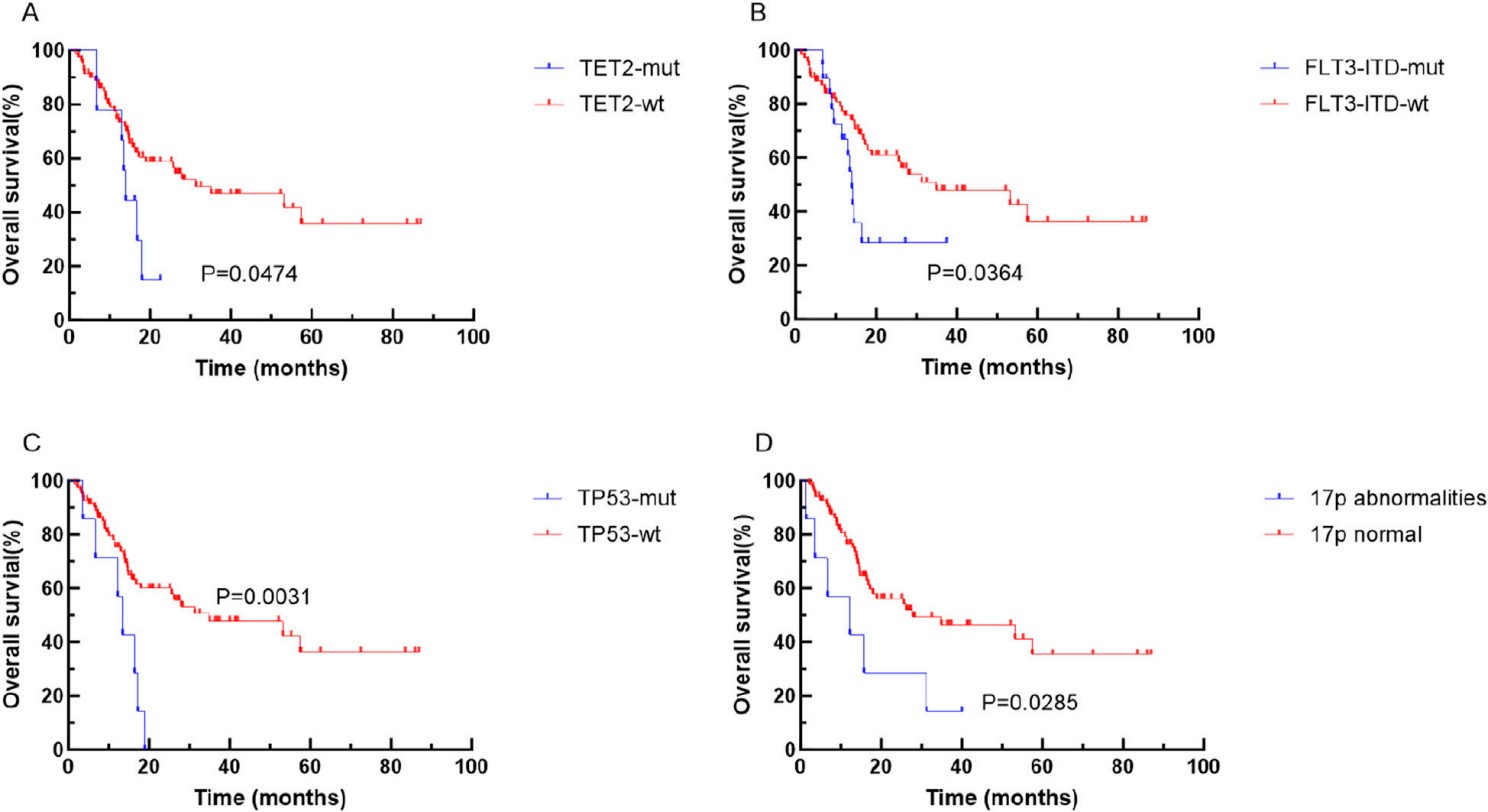
Kaplan-Meier curves for survival of patients with or without specific genetic mutations in elderly AML. The red and blue lines represent the survival of patients without or with mutations, abnormalities or normal chromosome karyotypes respectively. **(A)** OS for patients with or without *TET2* mutation (p = 0.0474). **(B)** OS for patients with or without *FLT3-ITD* mutation (p = 0.0364). **(C)** OS for patients with or without *TP53* mutation (p = 0.0031). **(D)** OS for patients abnormalities or normal 17P (p < 0.001). Abbreviations: OS, overall survival; wt, wild type; mut, mutant.

**FIGURE 3:**
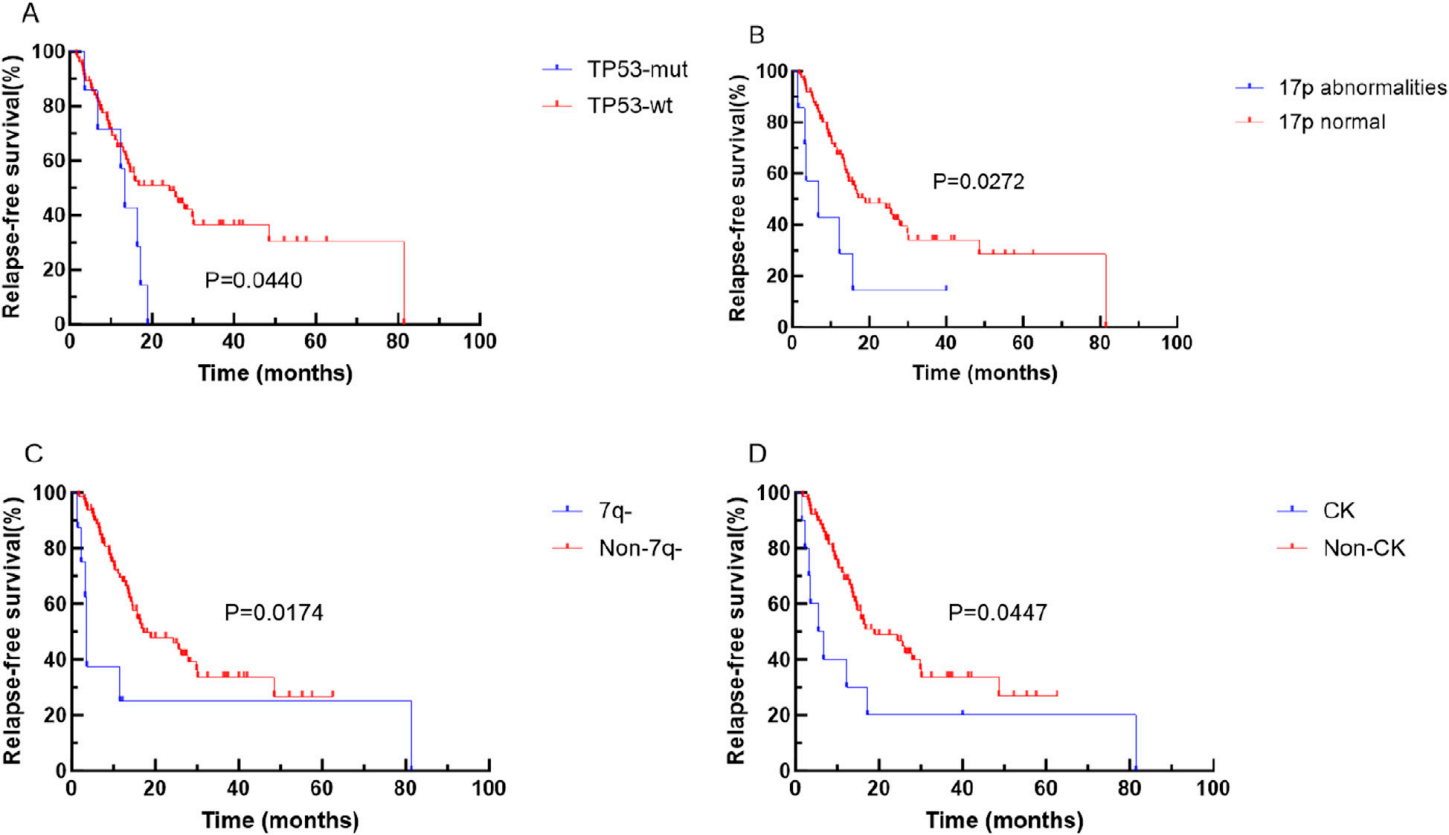
Kaplan-Meier curves for survival of patients with or without specific genetic mutations in elderly AML. The red and blue lines represent the survival of patients without or with mutations, abnormalities or normal chromosome karyotypes respectively. **(A)** RFS for patients with or without TP53 mutation (p = 0.0440). **(B)** RFS for patients abnormalities or normal 17P (p = 0.0272). **(C)** RFS for patients abnormalities or normal 7q (p = 0.0174). **(D)** RFS for patients with or without CK (p = 0.0447). Abbreviations: RFS, relapse-free survival; wt, wild type; mut, mutant.

Kaplan-Meier analysis unveiled that specific genetic mutations and chromosomal abnormalities were associated with shorter OS and RFS. Notably, *TET2* mutation (P = 0.0474), *FLT3-ITD* mutation (P = 0.0364), *TP53* mutation (P = 0.0031), and 17p abnormality (P = 0.00285) were predictive of shorter OS (refer to Figure 2). Additionally, *TP53* mutations (P = 0.0440), 17 abnormality (P = 0.0272), 7q abnormality (P = 0.0174), and CK (P = 0.0447), were predictive of shorter RFS (see Figure 3).

### Multivariate analysis of prognosis in elderly patients with AML

In the univariate analysis, several factors, including achieving CR, presence of *TET2* mutation, *FLT3-ITD* mutation, *TP53* mutation, and 17p abnormality, were observed to significantly influence the prognosis of elderly AML patients. To further delve into multiple factor analysis, the investigation aimed to ascertain whether induction chemotherapy effectiveness and the presence of 17p abnormalities independently served as prognostic factors.

Following multivariate analysis for RFS, it was identified that the presence of 7q-abnormality emerged as an independent prognostic factor (as indicated in Table 4). This suggested that, among the various factors assessed, 7q-abnormality specifically stood out as an independent indicator influencing the prognosis of elderly AML patients in terms of RFS. However, the analysis did not identify induction chemotherapy effectiveness or the presence of 17p abnormalities as independent prognostic factors in this specific context.

**TABLE 4:** Multivariate analysis for survival of new diagnosed elderly AML patients.

Factors	HR (95% CI)	p value
OS		
Response to chemotherapy (NR vs. CR)	0.154 (0.074–0.321)	<0.001
17p (normal vs. abnormal)	2.714 (1.012–7.279)	0.047
RFS		
7q- (normal vs. abnormal)	3.166 (1.159–8.648)	0.025

## Discussion

The initial treatment of AML necessitates a comprehensive evaluation encompassing cytogenetic and molecular genetic assessments, which play pivotal roles in stratifying prognosis and tailoring treatment strategies. In the context of elderly patients with AML (excluding M3 subtype), this demographic represents a distinct group with specific challenges. Often, these individuals exhibit reduced tolerance to conventional cytotoxic chemotherapy and hematopoietic stem cell transplantation (Jaiswal and Ebert, 2019). Additionally, as age progresses, there is an increased incidence of adverse genetic mutations, CK, MK, and other unfavorable outcomes (Creutzig et al., 2016). Given these challenges and the unique characteristics of AML in the elderly, our study aimed to gather nearly 7 years’ worth of clinical data from elderly AML patients. Through a comprehensive analysis of their biological characteristics, including cytogenetics and molecular genetics, the goal was to assess prognosis and provide guidance for clinical practices. By thoroughly examining these biological markers and their impact on prognosis, our study aspired to offer valuable insights to aid in better understanding the disease and in guiding more effective clinical approaches for this specific patient population.

Cytogenetics plays a crucial role in predicting the prognosis of patients diagnosed with AML. Structural distortions observed in cytogenetic analysis often signify a poor prognosis for AML patients (Harrison et al., 2010). In our study involving newly diagnosed elderly AML patients, 22.2% exhibited a CK, a factor commonly associated with a poor prognosis. This finding was consistent with the incidence observed internationally (Daneshbod et al., 2019). Among the subset of patients with CK, 50% achieved CR, aligning with global data trends (Daneshbod et al., 2019). However, it is essential to note that the definition of CK varies across classifications. Our study did not find CK (defined as ≥3 unrelated chromosomal abnormalities) to be predictive of OS or RFS in elderly AML patients. Interestingly, highly abnormal CK was linked to a lower RFS in this patient group. Regarding MK, our study identified its presence in 8.9% of patients, a proportion notably lower than the 20% reported in foreign studies (Medeiros et al., 2010). This discrepancy led us to hypothesize that variations might be related to factors such as the racial composition of the study population (Yi et al., 2020). Deletions in chromosome arms 5q-, 7q-, or 17p are indicative of poor prognosis in AML. However, in our study, while structural aberrations involving 5q-, 7q-, or 17p deletions did not significantly affect the CR rate in elderly AML patients, 17p deletions predicted lower OS or RFS, and 7q-deletions predicted lower OS. Surprisingly, 5q-aberrations did not show predictive value for OS or RFS in elderly AML patients in our study.

Gene mutations play a significant role in determining the prognosis of patients diagnosed with AML. While guidelines like NCCN (Pollyea et al., 2021) emphasize the prognostic impact of certain mutations in adult AML patients, their influence on the prognosis of elderly AML patients warrants further investigation. Notably, mutations in genes such as *FLT3-ITD*, *ASXL1*, *TP53*, *NPM1*, *CEBPA*, and *TET2* carry prognostic implications, albeit with varying effects in elderly AML patients compared to adult populations. *TP53* mutations, a factor associated with poor tumor survival, have been consistently linked to lower OS or CR rates in AML patients (Zhang et al., 2017; Xu et al., 2022). In our study of elderly AML patients, *FLT3-ITD* mutations were the most prevalent (21.1%), aligning with previous reports (Daneshbod et al., 2019). Interestingly, these patients exhibited lower CR rates and reduced OS, in line with findings by other researchers (Daver et al., 2021). Despite targeted drugs for *FLT3-ITD*, the prognosis remains poor with a high recurrence rate, often necessitating early allo-HSCT (allogeneic hematopoietic stem cell transplantation) for eligible patients to potentially improve outcomes (Khanolkar et al., 2022). Contrary to established views, the presence of *NPM1* and/or *CEBPA* double mutations did not significantly impact CR or OS in elderly AML patients in our study (Yang et al., 2020). This contrasts with prior research, and we hypothesize that the discrepancy could be attributed to the patient group’s composition, wherein genetic mutations or other poor prognosis indicators may interact differently within abnormal chromosomal karyotypes. Recent studies have pointed towards *TET2* mutations as predictive markers for lower OS or RFS in AML patients (Sasaki et al., 2020; Wang et al., 2020), which our study supported, indicating lower OS in elderly AML patients with *TET2* mutations. Conversely, while patients with *ASXL1* mutations displayed lower OS rates in our study, this mutation alone did not predict OS or RFS, differing from the findings of other researchers (Fan et al., 2021). This discrepancy may be attributed to the interplay of *ASXL1* mutations with other adverse prognosis gene mutations in patients lacking *ASXL1* mutations. In our investigation, elderly AML patients exhibited distinctive genetic characteristics, showcasing a predisposition towards gene mutations and chromosomal abnormalities. Typically, *NPM1* and/or *CEBPA* mutations signify a favorable prognosis in AML patients. However, intriguingly, in this study involving elderly patients, these gene mutations did not seem to impact prognosis significantly. Instead, the presence of 17p abnormality emerged as an independent prognostic factor affecting OS, while 7q-abnormalities played a role in predicting RFS in these AML patients.

The study sheds light on the prognostic evaluation of elderly AML patients, though it is constrained by the retrospective nature and the limited number of studies. In the future, we anticipate more clinical studies will be carried out to develop prognostic models, improving the evaluation and treatment of elderly AML patients.

## Conclusion

The overall prognosis for elderly individuals with AML poses greater challenges compared to adults. And favorable prognosis genes do not seem to apply to elderly AML patients. The complexity arises from various factors, including a higher prevalence of genetic mutations and chromosomal anomalies in this age group.

Integrating cytogenetic and molecular genetic information becomes pivotal to offer better insights for treatment strategies and prognosis assessment. Enhancing the evaluation system for prognosis in elderly AML patients is crucial. By refining our understanding of the interplay between genetic mutations, chromosomal abnormalities, and their implications for prognosis, we aim to facilitate more informed clinical decision-making. It is our hope that this study contributes to the enhancement of prognostic evaluation systems for elderly AML patients, ultimately aiding in guiding more effective and personalized clinical approaches for this specific patient population.

## Data Availability

The original contributions presented in the study are included in the article/supplementary material, further inquiries can be directed to the corresponding author.

## Abbreviations

CR, remission; NR, no remission; OS, overall survival; Wt, wild type; Mut, mutant; RFS, relapse-free survival; BM, bone marrow; ELN, European Leukemia Net.
